# High Charge Carrier Mobility in Non‐Conjugated 3D Covalent Organic Frameworks

**DOI:** 10.1002/adma.202517211

**Published:** 2026-02-03

**Authors:** Joaquín Almarza, Karol Strutyński, Shuai Fu, Yifan Chen, Natalia M. Padial, Isabel del Barrio Gómez, Carlos Martí‐Gastaldo, Andrei N. Khlobystov, Mischa Bonn, Hai I. Wang, Manuel Melle‐Franco, Aurelio Mateo‐Alonso

**Affiliations:** ^1^ POLYMAT Department of Applied Chemistry University of the Basque Country UPV/EHU Donostia‐San Sebastián Spain; ^2^ CICECO Aveiro Institute of Materials Department of Chemistry University of Aveiro Aveiro Portugal; ^3^ Max Planck Institute For Polymer Research Mainz Germany; ^4^ School of Chemistry University of Nottingham Nottingham UK; ^5^ Instituto De Ciencia Molecular Universidad De Valencia Paterna Spain; ^6^ Departamento de Química Inorgánica Universidad de Granada Avenida Fuentenueva S/N, Granada Spain; ^7^ Nanophotonics Debye Institute for Nanomaterials Science Utrecht University Utrecht The Netherlands; ^8^ Ikerbasque Basque Foundation for Science Bilbao Spain

**Keywords:** 3D COFs, charge transport, non‐planar aromatics

## Abstract

π‐Conjugation, π–π stacking, and long‐range order play a central role in determining charge transport efficiency in organic materials. Covalent organic frameworks (COFs), which uniquely integrate these key structural and electronic features, have emerged as a promising platform for the development of novel electrically conductive organic materials. Intraframework π‐conjugation has shown to play a key role in improving charge carrier mobility. Here, we report **Joa‐COF‐2**, a 3D COF lacking extended intraframework π‐conjugation, yet exhibiting a remarkable charge carrier mobility of 14 ± 1  cm^2^ V^−1^ s^−1^. **Joa‐COF‐2** adopts an 11‐fold interpenetrated *dia* topology, featuring a tight interframework π–π stacking, which enables efficient charge transport in the absence of intraframework π‐conjugation.

## Graphical abstract

A non‐conjugated 3D COF constructed via boronate ester linkages from tetraphenylene and pyrene‐based monomers is reported, which exhibits a high charge carrier mobility of 14 ± 1 cm^2^ V^−1^ s^−1^.

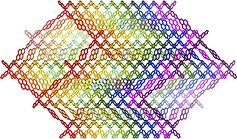

## 1 Introduction

π‐Conjugation, π–π stacking, and long‐range molecular order are essential for achieving efficient charge transport in organic materials [1, 2]. π‐Conjugation enables intramolecular charge transport, increases carrier delocalization, lowers energetic barriers, and allows tuning energy levels. In comparison, π–π stacking enables intermolecular charge transport through orbital overlaps between adjacent molecules. Lastly, long‐range order enables coherent transport mechanisms, leading to more efficient charge propagation and higher carrier mobilities. For instance, high mobilities (>1 cm^2^ V^−1^ s^−1^) are typically found in highly ordered organic systems (e.g., single crystals or ordered films) [1].

Covalent organic frameworks (COFs) –crystalline polymers built from covalently linked organic monomers– are particularly promising for the development of novel conducting materials, as they integrate all key factors that facilitate efficient charge transport, including π‐conjugation, π–π stacking, and long‐range structural order [3, 4]. At the monomer level, π‐conjugated monomers can be designed and introduced to confer specific structural characteristics, as well as tailored optoelectronic and electrical properties to the COFs. At the COF level, intraframework pathways for charge transport can be established or disrupted by a careful selection of the linkages (e.g., π‐conjugated, non‐conjugated) between monomers. Additionally, interframework charge transport pathways can arise from π–π stacking between different frameworks. Furthermore, the inherent crystallinity of COFs favors coherent transport mechanisms.

Studies on 2D COFs have shown that π‐conjugation plays a key role in improving charge carrier mobility [5, 6, 7, 8, 9, 10, 11, 12, 13, 14, 15]. π‐Conjugated 2D COFs typically incorporate π‐extended monomers (e.g., pyrene, phthalocyanine, coronene) connected through π‐conjugated linkages (e.g., imine, cyanostilbene, pyrazine), resulting in extended π‐conjugation that facilitates intralayer charge transport. Additionally, the use of π‐extended monomers promotes π–π stacking between layers, enabling efficient interlayer charge transport. These advances in π‐conjugated 2D COFs have motivated the design and synthesis of π‐conjugated 3D COFs, which, despite the limited examples available due to synthetic challenges, have shown promising charge carrier mobilities of up to 2.7 cm^2^ V^−1^ s^−1^ [16, 17, 18].

**FIGURE 1:**
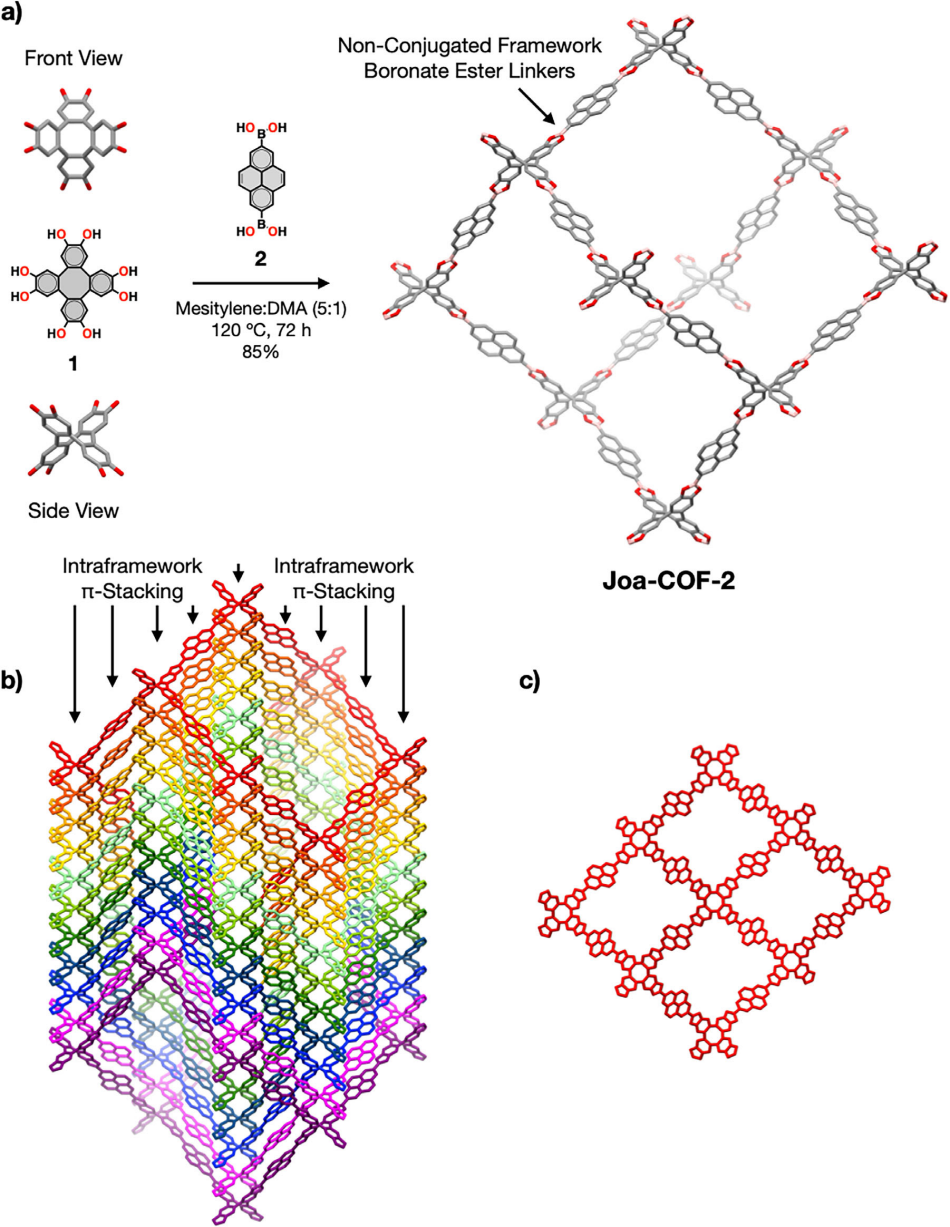
(a) Synthesis and reconstructed crystal structure of a single framework of **Joa‐COF‐2**. (b) Side and (c) top views of the reconstructed crystal structure of the fully interpenetrated *dia‐c11* **Joa‐COF‐2**, highlighting the interframework π–π stacking and the square‐shaped pores visible along the top‐down perspective.

Herein, we report the synthesis of a 3D COF (**Joa‐COF‐2**) built from non‐conjugated linkers and lacking extended intraframework π‐conjugation, yet exhibiting a remarkable charge carrier mobility of 14 ± 1  cm^2^ V^−1^ s^−1^ at room temperature. **Joa‐COF‐2** is constructed via boronate ester linkages by copolymerizing tetrahedral octahydroxy‐tetraphenylene nodes with pyrene diboronic acid struts (Figure 1). **Joa‐COF‐2** shows an 11‐fold interpenetrated *dia* topology, featuring a tight interframework π–π stacking. This arrangement opens up high mobility charge transport channels through strong electronic coupling between the stacked, interpenetrated frameworks.

## 2 Results and Discussion

In 2019, some of us introduced the use of non‐planar aromatics as nodes in COFs, as a strategy to access novel frameworks with non‐standard geometries that deviate from those typically dictated by sp, sp^2^, and sp^3^ carbon hybridizations [19, 20, 21, 22, 23]. This approach enabled the formation of highly complementary 3D topologies that promote interpenetration by interframework π–π stacking [20]. Following this strategy, we decided to synthesise **Joa‐COF‐2** to explore the influence of tetraphenylene nodes in interframework interactions. The synthesis of **Joa‐COF‐2** requires the copolymerisation of 2,3,6,7,10,11,14,15‐octahydroxytetraphenylene (**1**) [24] and pyrene‐2,7‐diboronic acid (**2**) [25] (Figure 1) that were synthesised following reported methods. **Joa‐COF‐2** was obtained by solvothermal synthesis by the condensation between tetraphenylene **1** and pyrenediboronic acid **2** in mesitylene/*N*,*N*‐dimethylacetamide (5:1, v:v) at 125°C for 3 days in a sealed ampoule, affording a white solid (85% yield) upon filtration and solvent washings. Importantly, tetraphenylene **1** was freshly prepared and used immediately prior to the synthesis of **Joa‐COF‐2**, as tetraphenylene **1** tends to decompose over time.

**FIGURE 2:**
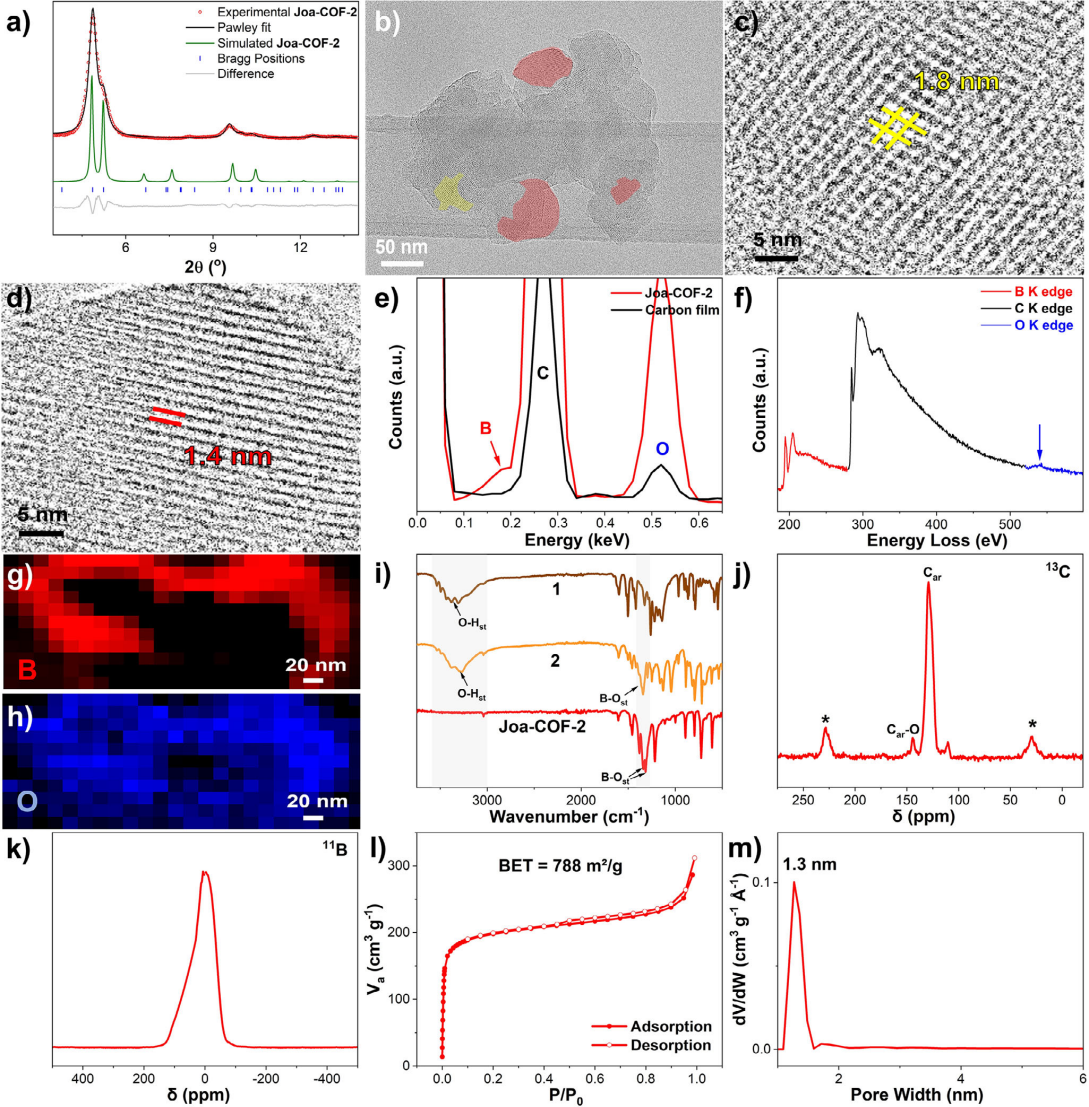
Structural, spectroscopic, and gas sorption characterization of **Joa‐COF‐2**. (a) Experimental and simulated PXRD patterns. (b–d) TEM images. The yellow area shows 1.8 nm lattice fringes, red area shows 1.4 nm lattice fringes. (e) EDX spectra of **Joa‐COF‐2** and carbon film. (f) Background‐subtracted EEL spectrum showing the B K‐edge, C K‐edge, and O K‐edge. EELS maps signal from (g) B and (h) O. (i) FT‐IR spectra. (j) ^1^ ^3^C and (k) ^11^B SS CP/MAS NMR. Rotation bands are marked with asterisks. (l) Nitrogen adsorption and desorption isotherm at 77 K. (m) DFT pore size distribution.

Powder X‐ray diffraction (PXRD) of **Joa‐COF‐2** shows several resolved reflections illustrating its crystalline nature (Figure 2a). The atomic structure of **Joa‐COF‐2** was elucidated by comparing the simulated PXRD patterns of different computed crystal structures with the experimental ones. Crystal structures were explored with a Tight‐Binding (TB) Hamiltonian and refined with DFT (PBE‐MBD/light) with DFTB+ [26, 27] and FHI‐aims [28, 29, 30], respectively, to produce the final structures (full details in Supporting Information). Given the symmetry and fourfold valency of octahydroxytetraphenylene **1**, a *T*_d_ tetragonal lattice with a (2,4)‐connected *dia* topology was constructed for **Joa‐COF‐2**, in which the octahydroxytetraphenylene nodes are condensed to the pyrenediboronic acid linkers by four boronate esters (Figure 1a). On this basis, among different levels of interpenetration (Figure S1), the 11‐fold interpenetrated (*dia‐c11*) **Joa‐COF‐2** structure was found to best reproduce the experimental PXRD diffractogram, where the peaks at 4.9°, 5.1°, 9.6° and 10.4° correspond to the (110), (1$\bar{1}0$), (220) and (2$\bar{2}$0) reflections, respectively (Figure 2a; Figure S2). We have refined the corresponding cell parameters with the Pawley procedure (Figure 2a; Table S1), confirming the validity of the structure. Interpenetration is strongly favoured in these COFs through interframework π–π stacking (3.45 Å between pyrenes), where the tetraphenylene nodes sit on top of each other in a cofacial AA arrangement (Figure 1b).

The crystallinity of **Joa‐COF‐2** is also confirmed by high‐resolution transmission electron microscopy (HRTEM). The measurements were conducted under low‐dose imaging conditions using a 200 keV electron beam and a Gatan K3 IS single‐electron detector camera. This approach enabled detailed imaging of the COF structure while minimising electron beam damage. Large‐field‐of‐view images revealed polycrystalline particles consisting of different crystalline domains (Figure 2b). Close inspection of these domains identified two prominent lattice fringes at 1.8 and 1.4 nm (Figure 2c,d). The 1.8 nm spacing observed in two sets of intersecting planes (Figure 2c; Figure S3) corresponds to the (110) and (1$\bar{1}0$) lattice planes of *dia‐c11* **Joa‐COF‐2**. The angle between the planes (78°) is consistent with the theoretically predicted (82°). Whereas the observed 1.4 nm lattice spacing arises from the stacked pyrene columns (Figure 2d; Figure S3) and corresponds to the (020) lattice plane. Energy‐dispersive X‐ray (EDX) analysis of a COF particle revealed the presence of carbon, oxygen, and boron, with the boron peak overlapping the strong carbon peak, yet remaining distinguishable (Figure 2e). Electron energy loss spectroscopy (EELS) confirmed the presence of all the elements (Figure 2f), and the distribution of boron and oxygen in the scanning transmission electron microscopy‐EELS maps correlated with the position of the COF particle (Figure 2g,h). The B signal is observable in the thin edge of the particle where single‐scattering events are prevalent. To ensure that the oxygen signal from the amorphous carbon film is not a concern, the EELS data were acquired from the region where the sample is hanging over the vacuum.

The structure of **Joa‐COF‐2** is also consistent with spectroscopic and gas sorption characterisation. The FT‐IR spectra of **Joa‐COF‐2** confirmed the condensation of monomers **1** and **2** with the appearance of a strong C─O stretching band at 1350 and 1306 cm^−1^, typical of boronate esters, and a strong attenuation of O─H stretching band of the hydroxyl groups present on the monomers (Figure 2i). The solid‐state cross‐polarization magic angle spinning (SS CP/MAS) ^13^C NMR signals of **Joa‐COF‐2** are consistent with the aromatic nature of tetraphenylene and pyrene moieties (Figure 2j); meanwhile, the SS CP/MAS ^11^B NMR spectrum shows a peak consistent with the presence of the boronate ester linkages (Figure 2k). The ^1^H NMR spectrum of **Joa‐COF‐2** incubated in DMSO‐*d*_6_ shows the signals of the hydrolysed tetraphenylene **1** and of pyrene diboronic acid **2** in a 1:2 ratio in agreement with the expected stoichiometry (Figure S4). The pore size distribution of **Joa‐COF‐2** was experimentally investigated by nitrogen adsorption measurements at 77 K. **Joa‐COF‐2** displays a reversible type IV isotherm with both microporous and mesoporous regions, and a Brunauer–Emmett–Teller (BET) surface area of 756 m^2^ g^−1^ (Figure 2l). The pore‐size distribution plot generated by fitting the experimental adsorption isotherm using a Density Functional Theory (DFT) slit pore model shows a broad peak centred at 1.3 nm (Figure 2m). The deviation is small and matches the expected microporosity (Table S2). The non‐interpenetrated system would result in very large mesopores (>2 nm), whereas the experimental value is consistent with 11‐fold interpenetration and the resulting reduction in pore size.

**FIGURE 3:**
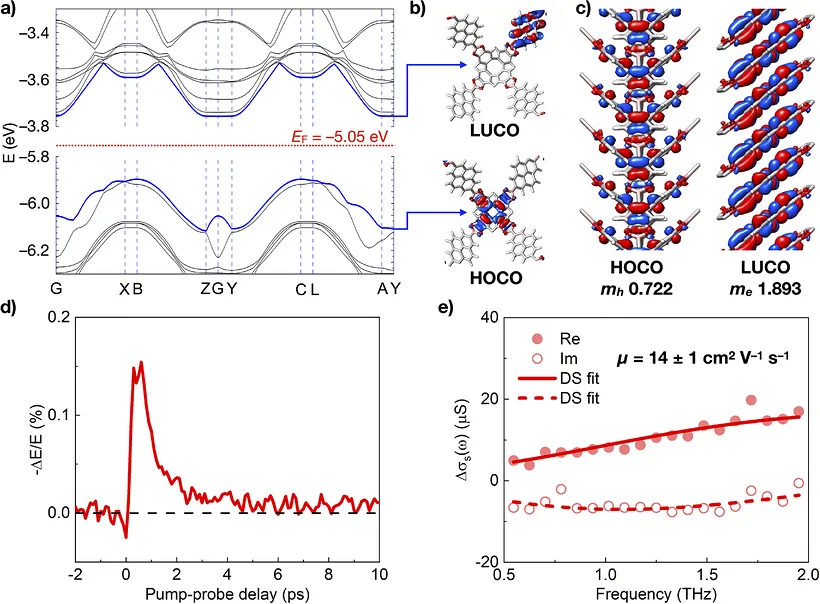
(a) Band structure of **Joa‐COF‐2**. (b) Top and (c) side views of the HOCO and LUCO of *dia‐c11* **Joa‐COF‐2**. (d) Time‐resolved THz photoconductivity of **Joa‐COF‐2**. The sample was photoexcited by a 3.1 eV femtosecond laser pulse with an incident pump fluence of 255 µJ/cm^2^. (e) Frequency‐resolved complex THz photoconductivity spectrum measured 0.5 ps after the peak photoconductivity. The solid and dashed lines represent the Drude‐Smith fits to the real and imaginary components of the complex THz photoconductivity, respectively.

To provide additional insights into the electronic structure and the charge transporting properties of the *dia‐c11* **Joa‐COF‐2** structure, we analysed the PBE/light‐computed electronic band structure (Figure 3), which agrees with the B3LYP/light‐computed band structure (Figure S5 and Table S3). The PBE/light indirect bandgap (2.14 eV, from B/C to G) is in close agreement with the optical bandgap (Figure S6). The Highest Occupied Crystalline Orbital (HOCO) is fundamentally located on tetraphenylene nodes and has a low hole mass (*m_h_* = 0.722 m_0_) (Figure 3a,b; Figures S7 and S8). The Lowest Unoccupied Crystalline Orbital (LUCO) is located on the pyrene struts and shows a heavier electron mass (*m_e_* = 1.893 m_0_) (Figure 3a,b; Figures S7 and S8). The HOCO and LUCO electron densities are highly delocalised over neighbouring tetraphenylenes and pyrenes, respectively, enabling channels for charge transport (Figure 3c).

The charge transport properties of **Joa‐COF‐2** were investigated using optical‐pump THz‐probe (OPTP) spectroscopy. In these measurements, charge carriers are photoexcited by 3.1 eV femtosecond laser pulses, and the resulting pump‐induced attenuation of the transmitted THz electric field (−Δ*E*/*E*), caused by free carrier absorption, is recorded. This relative change is linearly proportional to the transient photoconductivity *Δσ* [31]. As shown in Figure 3d, the photoexcitation results in a sub‐ps charge carrier generation (and thus rise in *Δσ)*, followed by a ps decay of *Δσ* due to trapping at defects or exciton formation [12, 13, 32]. We rule out radiative recombination as the dominant decay pathway because of the weak photoluminescence of the sample. Figure 3e shows the complex THz photoconductivity (Δσ(ω)) measured at 0.5 ps after the peak photoconductivity. We fit the obtained *Δσ*(ω) by the Drude‐Smith (DS) model (Equation 1), which phenomenologically describes spatially confined charge transport through a backscattering parameter *c* ranging between 0 (for isotropic scattering) and –1 (for preferential backscattering).

(1) $$\sigma_{DS}\left(\omega \right)=\frac{\omega_{p}^{2}\varepsilon_{0}\tau_{s}}{1-i\omega \tau_{s}}\cdot \left(1+\frac{c}{1-i\omega \tau_{s}}\right)$$

where ω*_p_*, *ε_0_*, and *τ_s_* represent the plasma frequency, vacuum permittivity, and Drude‐Smith scattering time, respectively. The best fit to the data yields *c* of –0.93 and *τ_s_* of 59 ± 4 fs.

To estimate the charge mobility (μ) of **Joa‐COF‐2** in the DC limit, Equation (2) was followed,

(2) $$\mu =\frac{e\tau_{s}}{m^{*}}\times \left(1+c\right)$$

where *e* is the elementary charge, and *m* ^*^ = 0.523 *m*_0_ is the reduced electron‐hole effective mass (obtained from $\frac{1}{m^{*}}=\frac{1}{m_{e}}+\frac{1}{m_{h}}$). This yields a charge mobility of 14 ± 1 cm^2^ V^−1^ s^−1^ in the DC limit. The obtained charge mobility value is very high, considering that **Joa‐COF‐2** is a non‐conjugated 3D COF, yet it is consistent with the interframework delocalization of electron density at the HOCO and the LUCO. These THz mobility values are in the same range as those observed in the best performing non‐conjugated 2D COFs, where charge transport occurs exclusively via vertical π–π stacking [9]. These observations indicate that electronic transport occurs preferentially across the channels formed by the tetraphenylene‐tetraphenylene and pyrene‐pyrene π‐stacks within the *dia‐c11* interpenetrated structure of **Joa‐COF‐2** (Figures 1b and 3c).

## 3 Conclusions

**Joa‐COF‐2** establishes a new benchmark for charge transport in 3D COFs, with a charge carrier mobility of 14 ± 1 cm^2^ V^−1^ s^−1^. This work demonstrates that high charge carrier mobility can be achieved in the absence of intraframework π‐conjugation, provided that close interframework π–π stacking is established through interpenetration. The 11‐fold interpenetrated *dia* topology of **Joa‐COF‐2**, combined with the tight interframework π–π stacking, opens efficient charge transport channels responsible for the high charge carrier mobility. These findings not only challenge the conventional design principles for conductive COFs but also open new avenues for engineering high‐mobility 3D frameworks through topological and supramolecular control rather than purely conjugated connectivity.

## Conflicts of Interest

The authors declare no conflicts of interest.

## Supporting information




**Supporting File 1**: adma72353‐sup‐0001‐SuppMat.pdf.

**Supporting File 2**: adma72353‐sup‐0002‐DataFile.zip.

## Data Availability

The data underlying this study is shown in the published article and its supporting information.

## References

[1] S. Fratini , M. Nikolka , A. Salleo , G. Schweicher , and H. Sirringhaus , “Charge Transport in High‐Mobility Conjugated Polymers and Molecular Semiconductors,” Nature Materials 19 (2020): 491–502, 10.1038/s41563-020-0647-2. 32296138

[2] Electronic Processes in Organic Semiconductors, (Wiley‐VCH Verlag GmbH & Co. KGaA, 2015).

[3] D. Jiang , V. G. W. Tan , Y. Gong , et al., “Semiconducting Covalent Organic Frameworks,” Chemical Reviews 125 (2025): 6203–6308. 40366230 10.1021/acs.chemrev.4c00950

[4] M. Martínez‐Abadía and A. Mateo‐Alonso , “Structural Approaches to Control Interlayer Interactions in 2D Covalent Organic Frameworks,” Advanced Materials 32 (2020): 2002366, 10.1002/adma.202002366. 32864762

[5] S. Wan , F. Gándara , A. Asano , et al., “Covalent Organic Frameworks with High Charge Carrier Mobility,” Chemistry of Materials 23 (2011): 4094–4097, 10.1021/cm201140r.

[6] X. Ding , J. Guo , X. Feng , et al., “Synthesis of Metallophthalocyanine Covalent Organic Frameworks that Exhibit High Carrier Mobility and Photoconductivity,” Angewandte Chemie International Edition 50 (2011): 1289–1293, 10.1002/anie.201005919. 21290495

[7] X. Feng , L. Liu , Y. Honsho , et al., “High‐Rate Charge‐Carrier Transport in Porphyrin Covalent Organic Frameworks: Switching from Hole to Electron to Ambipolar Conduction,” Angewandte Chemie International Edition 51 (2012): 2618–2622, 10.1002/anie.201106203. 22290932

[8] J. Guo , Y. Xu , S. Jin , et al., “Conjugated Organic Framework with Three‐Dimensionally Ordered Stable Structure and Delocalized π Clouds,” Nature Communications 4 (2013): 2736, 10.1038/ncomms3736. PMC3868157 24220603

[9] E. Jin , K. Geng , S. Fu , et al., “Exceptional Electron Conduction in Two‐Dimensional Covalent Organic Frameworks,” Chemistry 7 (2021): 3309–3324, 10.1016/j.chempr.2021.08.015.

[10] G. Xing , W. Zheng , L. Gao , et al., “Nonplanar Rhombus and Kagome 2D Covalent Organic Frameworks from Distorted Aromatics for Electrical Conduction,” Journal of the American Chemical Society 144 (2022): 5042–5050, 10.1021/jacs.1c13534. 35189061

[11] E. Jin , K. Geng , S. Fu , et al., “Module‐Patterned Polymerization towards Crystalline 2D sp^2^‐Carbon Covalent Organic Framework Semiconductors,” Angewandte Chemie International Edition 61 (2022): 202115020, 10.1002/anie.202115020. 34931425

[12] Y. Liu , S. Fu , D. L. Pastoetter , et al., “Vinylene‐Linked 2D Conjugated Covalent Organic Frameworks by Wittig Reactions,” Angewandte Chemie International Edition 61 (2022): 202209762, 10.1002/anie.202209762. PMC10098612 36161682

[13] S. Fu , E. Jin , H. Hanayama , et al., “Outstanding Charge Mobility by Band Transport in Two‐Dimensional Semiconducting Covalent Organic Frameworks,” Journal of the American Chemical Society 144 (2022): 7489–7496, 10.1021/jacs.2c02408. 35420808 PMC9052747

[14] R. Zhao , W. Wang , Y. Liu , et al., “A Donor–Acceptor‐Type Two‐Dimensional Poly(Arylene Vinylene) for Efficient Electron Transport and Sensitive Chemiresistors,” Angewandte Chemie International Edition 64 (2025): 202504302, 10.1002/anie.202504302. PMC12144878 40164570

[15] S. Fu , X. Li , G. Wen , et al., “Dimensional Evolution of Charge Mobility and Porosity in Covalent Organic Frameworks,” Nature Communications 16 (2025): 2219, 10.1038/s41467-025-57436-y. PMC11882946 40044681

[16] S. Wang , L. Da , J. Hao , et al., “A Fully Conjugated 3D Covalent Organic Framework Exhibiting Band‐Like Transport with Ultrahigh Electron Mobility,” Angewandte Chemie International Edition 60 (2021): 9321–9325, 10.1002/anie.202100464. 33527686

[17] I. Munoz‐Alonso , D. Bessinger , S. Reuter , et al., “Highly Crystalline and Oriented Thin Films of Fully Conjugated 3D‐Covalent Organic Frameworks,” Angewandte Chemie International Edition 64 (2025): 202505799. 10.1002/anie.202505799 PMC12363643 40518452

[18] S. Wang , X.‐X. Li , L. Da , et al., “A Three‐Dimensional sp^2^ Carbon‐Conjugated Covalent Organic Framework,” Journal of the American Chemical Society 143 (2021): 15562–15566, 10.1021/jacs.1c06986. 34533316

[19] M. Martínez‐Abadía , C. T. Stoppiello , K. Strutynski , et al., “A Wavy Two‐Dimensional Covalent Organic Framework from Core‐Twisted Polycyclic Aromatic Hydrocarbons,” Journal of the American Chemical Society 141 (2019): 14403–14410, 10.1021/jacs.9b07383. 31478367

[20] M. Martínez‐Abadía , K. Strutyński , B. Lerma‐Berlanga , et al., “π‐Interpenetrated 3D Covalent Organic Frameworks from Distorted Polycyclic Aromatic Hydrocarbons,” Angewandte Chemie International Edition 60 (2021): 9941–9946, 10.1002/anie.202100434. 33555658

[21] M. Martínez‐Abadía , K. Strutyński , C. T. Stoppiello , et al., “Understanding Charge Transport in Wavy 2D Covalent Organic Frameworks,” Nanoscale 13 (2021): 6829–6833, 10.1039/D0NR08962A. 33620062

[22] J. Almarza , I. Cardillo‐Zallo , K. Strutyński , et al., “Tubular Nanostructures from Large‐Pore 2D Covalent Organic Frameworks,” Angewandte Chemie International Edition 64 (2025): 202505935, 10.1002/anie.202505935. PMC12124442 40125869

[23] E. De Bolòs , S. Bera , K. Strutyński , et al., “Interlocked 2D Covalent Organic Frameworks from Overcrowded Nodes,” Journal of the American Chemical Society 147 (2025): 2579–2586, 10.1021/jacs.4c14453. 39803845 PMC12265044

[24] T. Ashirov , K. S. Song , and A. Coskun , “Salt‐Templated Solvothermal Synthesis of Dioxane‐Linked Three‐Dimensional Nanoporous Organic Polymers for Carbon Dioxide and Iodine Capture,” ACS Applied Nano Materials 5 (2022): 13711–13719.

[25] A. G. Crawford , Z. Liu , I. A. I. Mkhalid , et al., “Synthesis of 2‐ and 2,7‐Functionalized Pyrene Derivatives: an Application of Selective C—H Borylation,” Chemistry—A European Journal 18 (2012): 5022–5035, 10.1002/chem.201103774. 22415854

[26] B. Hourahine , B. Aradi , V. Blum , et al., “DFTB+, a Software Package for Efficient Approximate Density Functional Theory Based Atomistic Simulations,” The Journal of Chemical Physics 152 (2020): 124101, 10.1063/1.5143190. 32241125

[27] B. Aradi , B. Hourahine , and T. Frauenheim , “DFTB+, a Sparse Matrix‐Based Implementation of the DFTB Method,” The Journal of Physical Chemistry A 111 (2007): 5678–5684, 10.1021/jp070186p. 17567110

[28] V. Blum , R. Gehrke , F. Hanke , et al., “Ab Initio Molecular Simulations with Numeric Atom‐Centered Orbitals,” Computer Physics Communications 180 (2009): 2175–2196, 10.1016/j.cpc.2009.06.022.

[29] A. Marek , V. Blum , R. Johanni , et al., “The ELPA Library: Scalable Parallel Eigenvalue Solutions for Electronic Structure Theory and Computational Science,” Journal of Physics: Condensed Matter 26 (2014): 213201, 10.1088/0953-8984/26/21/213201. 24786764

[30] V. W. Yu , F. Corsetti , A. García , et al., “ELSI: a Unified Software Interface for Kohn–Sham Electronic Structure Solvers,” Computer Physics Communications 222 (2018): 267–285.

[31] R. Ulbricht , E. Hendry , J. Shan , T. F. Heinz , and M. Bonn , “Carrier Dynamics in Semiconductors Studied with Time‐Resolved Terahertz Spectroscopy,” Reviews of Modern Physics 83 (2011): 543–586, 10.1103/RevModPhys.83.543.

[32] M. Wang , S. Fu , P. Petkov , et al., “Exceptionally High Charge Mobility in Phthalocyanine‐Based Poly(benzimidazobenzophenanthroline)‐Ladder‐Type Two‐Dimensional Conjugated Polymers,” Nature Materials 22 (2023): 880–887, 10.1038/s41563-023-01581-6. 37337069 PMC10313522

