# Supplementary material for: High Charge Carrier Mobility in Non‐Conjugated 3D Covalent Organic Frameworks
Source: Adv Mater. 2026 Feb 3;38(51):e17211. doi: 10.1002/adma.202517211 (PMC13569066; doi:10.1002/adma.202517211)
Supplement: Supplementary file 1 [file ADMA-38-e17211-s002.pdf]

Supporting Information

High Charge Carrier Mobility in Non-Conjugated 3D Covalent Organic Frameworks

Joaquín Almarza, Karol Strutyński, Shuai Fu, Yifan Chen, Natalia M. Padial, Isabel del Barrio Gómez, Carlos Martí-Gastaldo, Andrei N. Khlobystov, Mischa Bonn, Hai I. Wang, Manuel Melle-Franco, and Aurelio Mateo-Alonso**

Table of Contents Page

Supplementary Figures 2
Supplementary Tables 10
Experimental Procedures 13
Synthetic Procedures 15
References 16

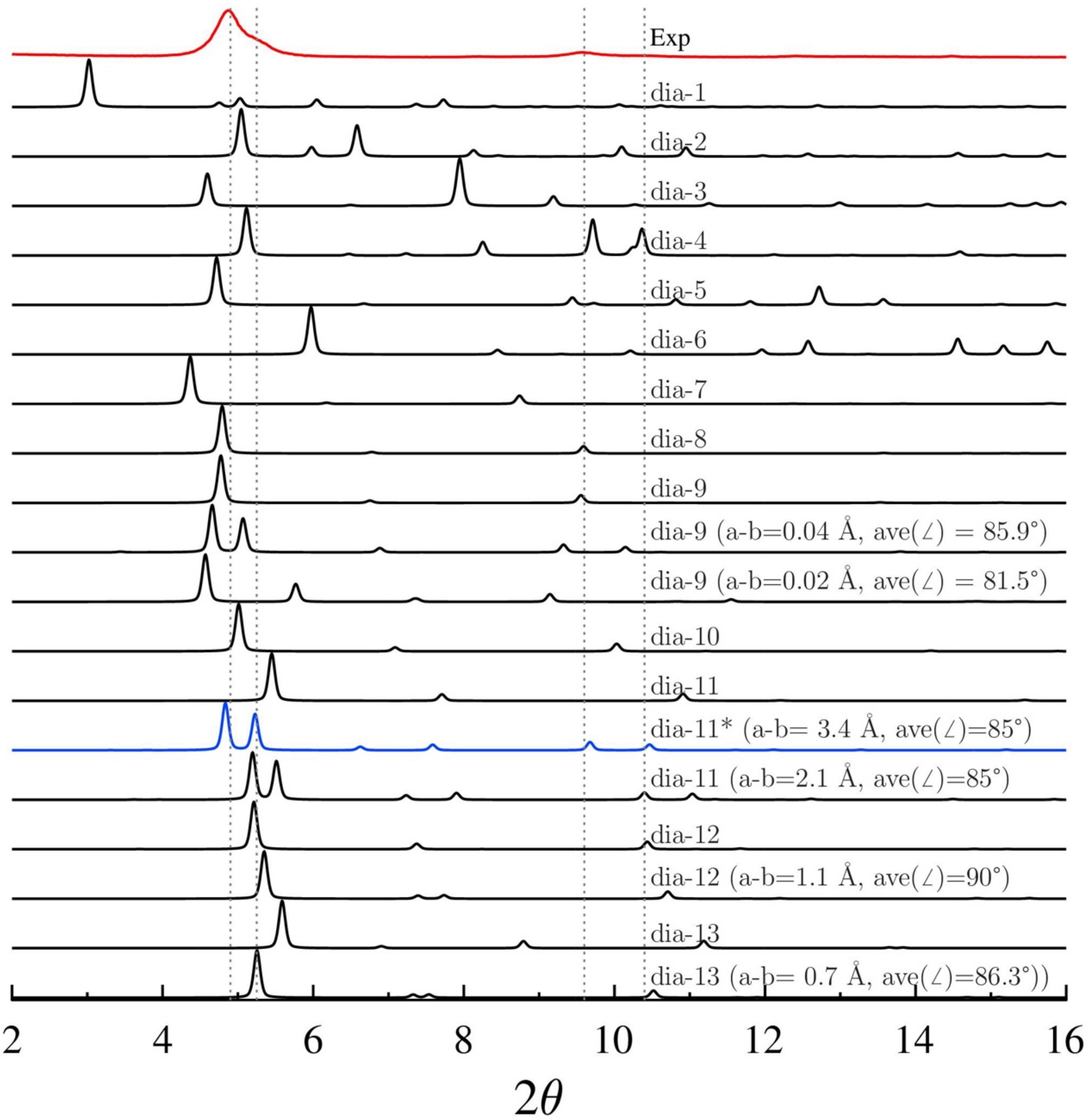

Figure S1. Simulated PXRD patterns of modelled tetragonal unit cells with different interpenetrations and selected asymmetric unit cells with different interpenetrations and geometries versus experiment (Exp), dia-c11* denotes the best fitting structure.

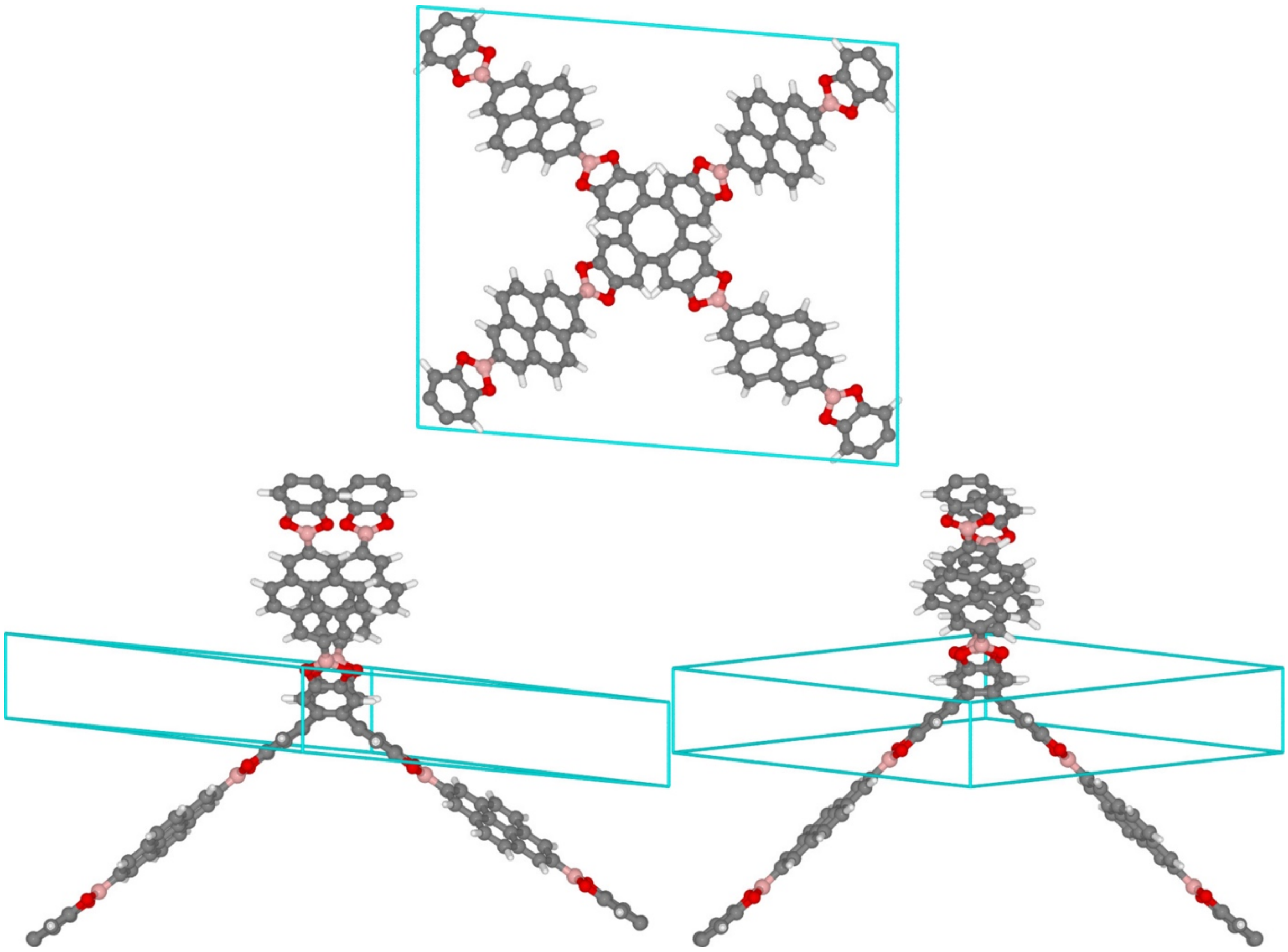

Figure S2. Views of the periodic unit cell in light-blue of *dia-c11* **Joa-COF-2**.

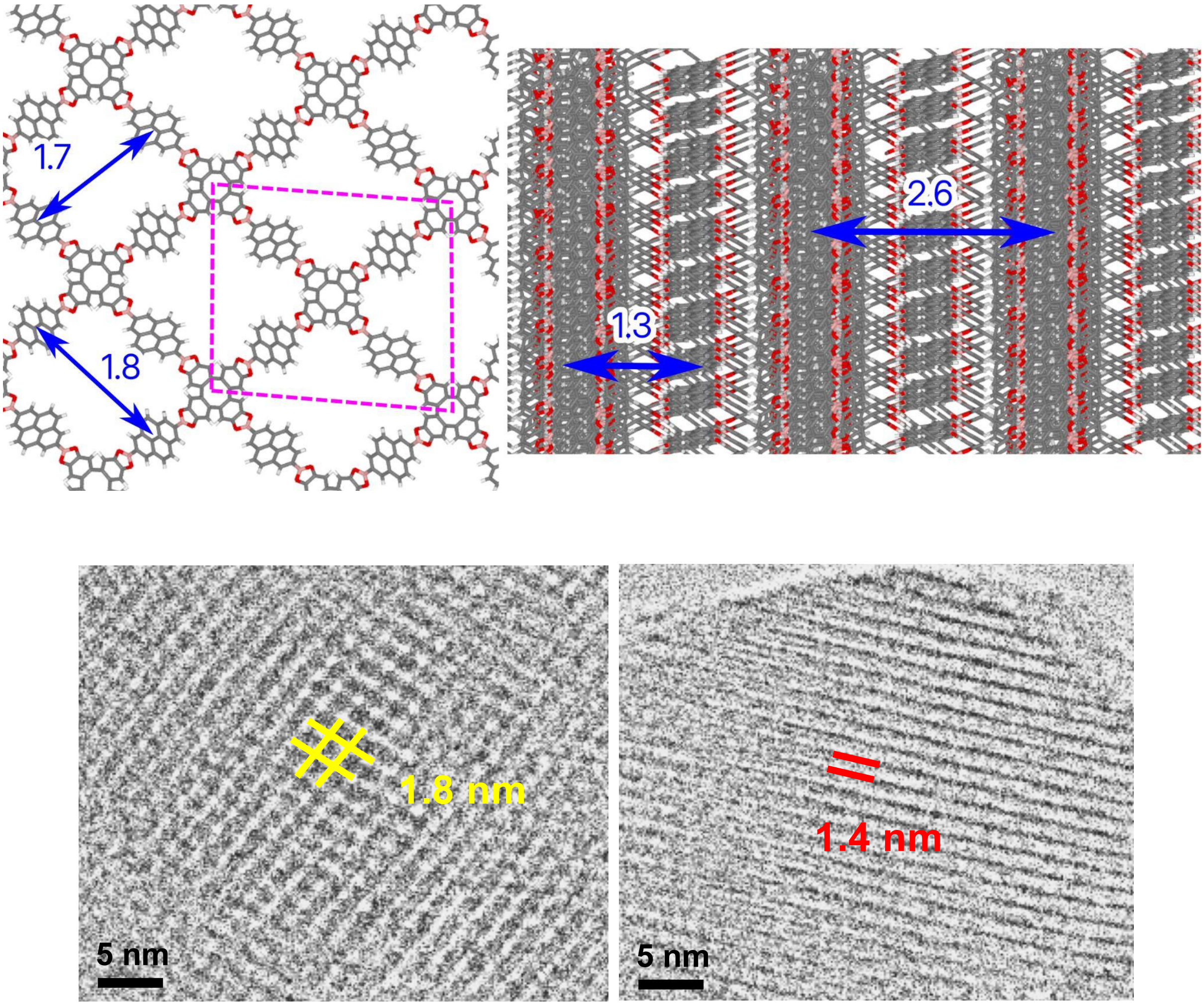

Figure S3. Distances between structural features of *dia-c11* **Joa-COF-2**.

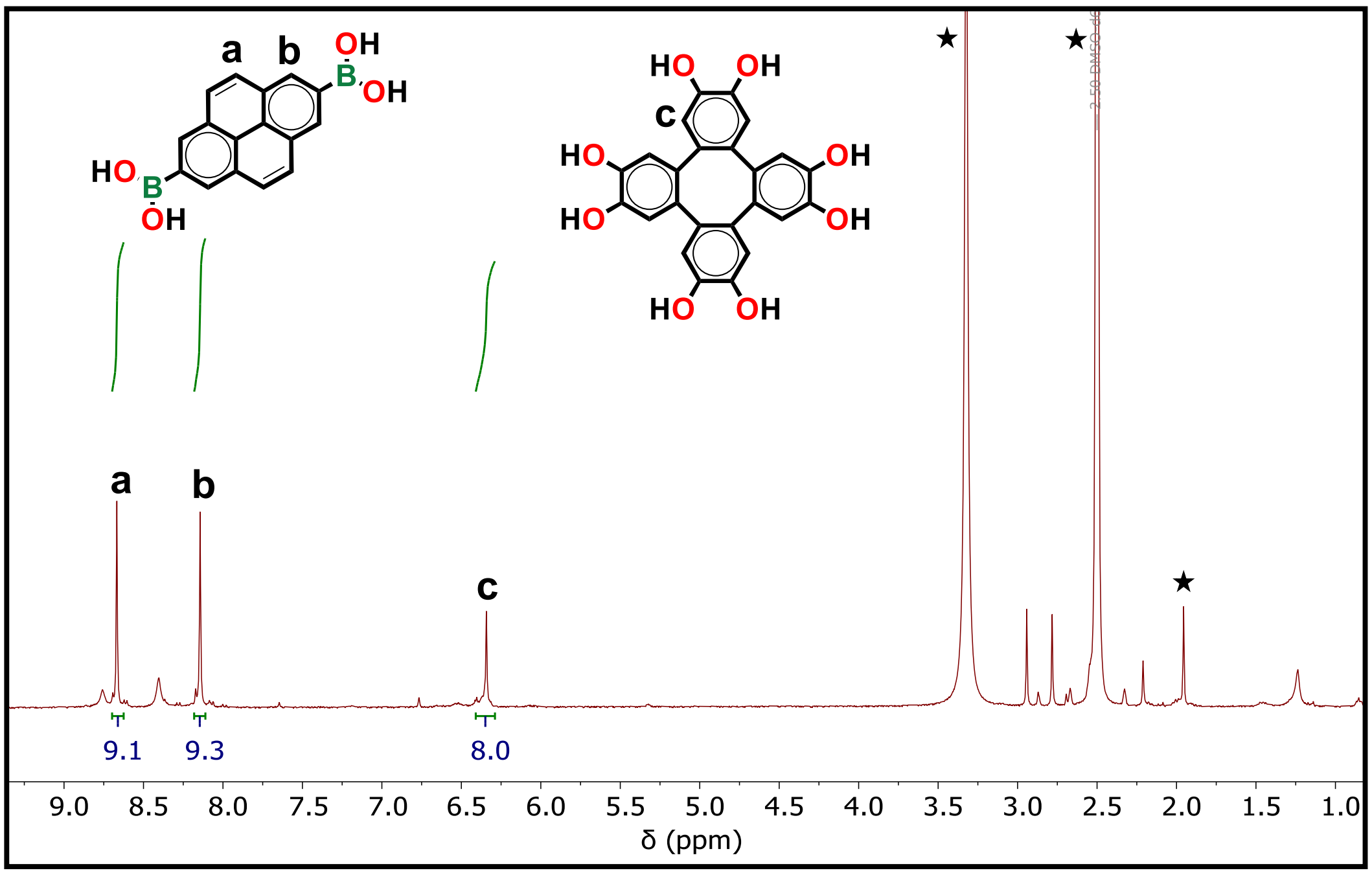

Figure S4. ^{1}H NMR of a 2 mg sample of **Joa-COF-2** dispersed in 0.6 mL DMSO-d_6 after incubation for 4 hours, showing signals of the hydrolysed tetraphenylene **1** and pyrene diboronic acid **2** in a 1:2 ratio (*indicates solvent residual peaks).

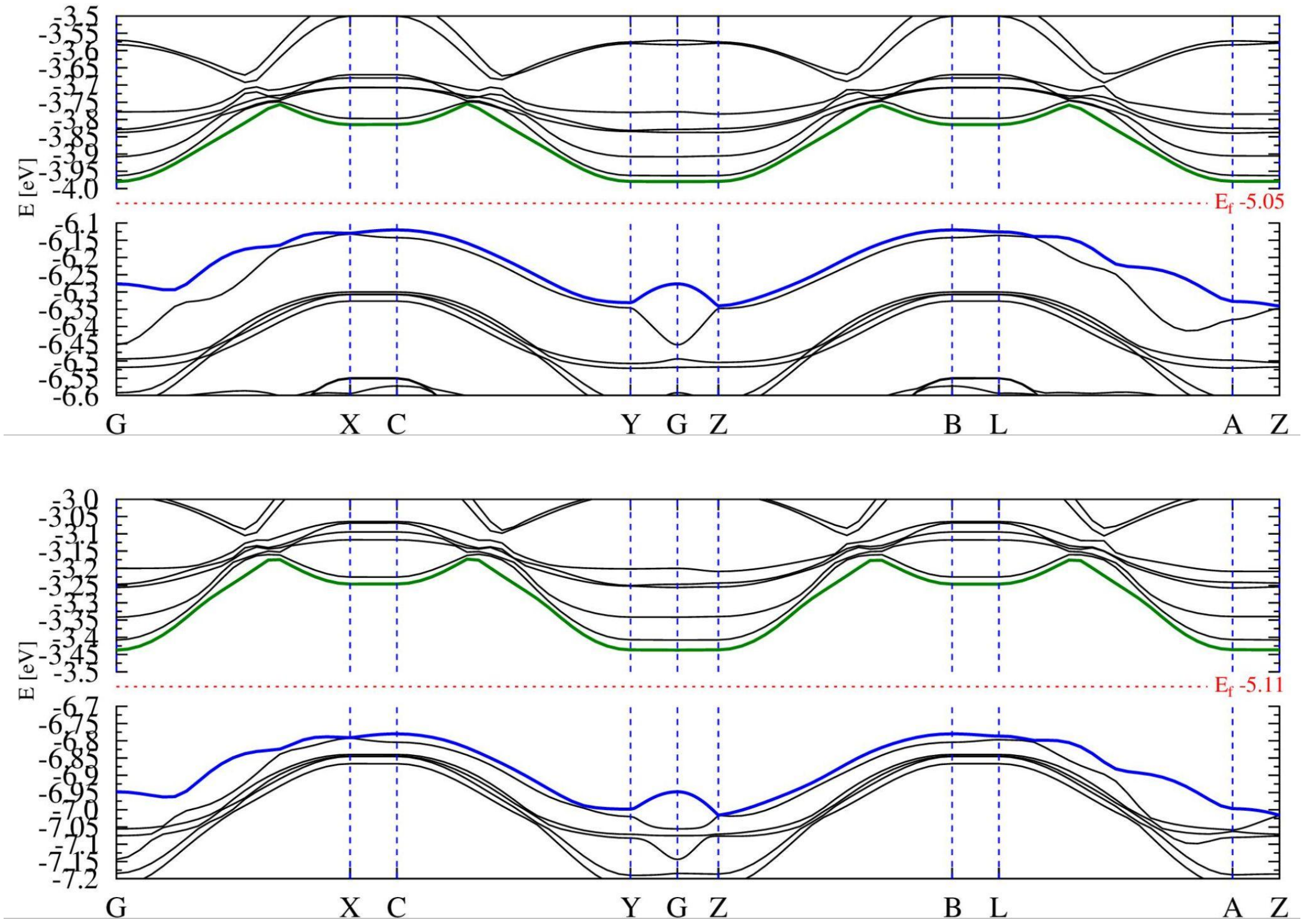

Figure S5. Comparison of the band structure of *dia-c11* **Joa-COF-2** computed with PBE/light (top) and with B3LYP/light (bottom) Hamiltonians.

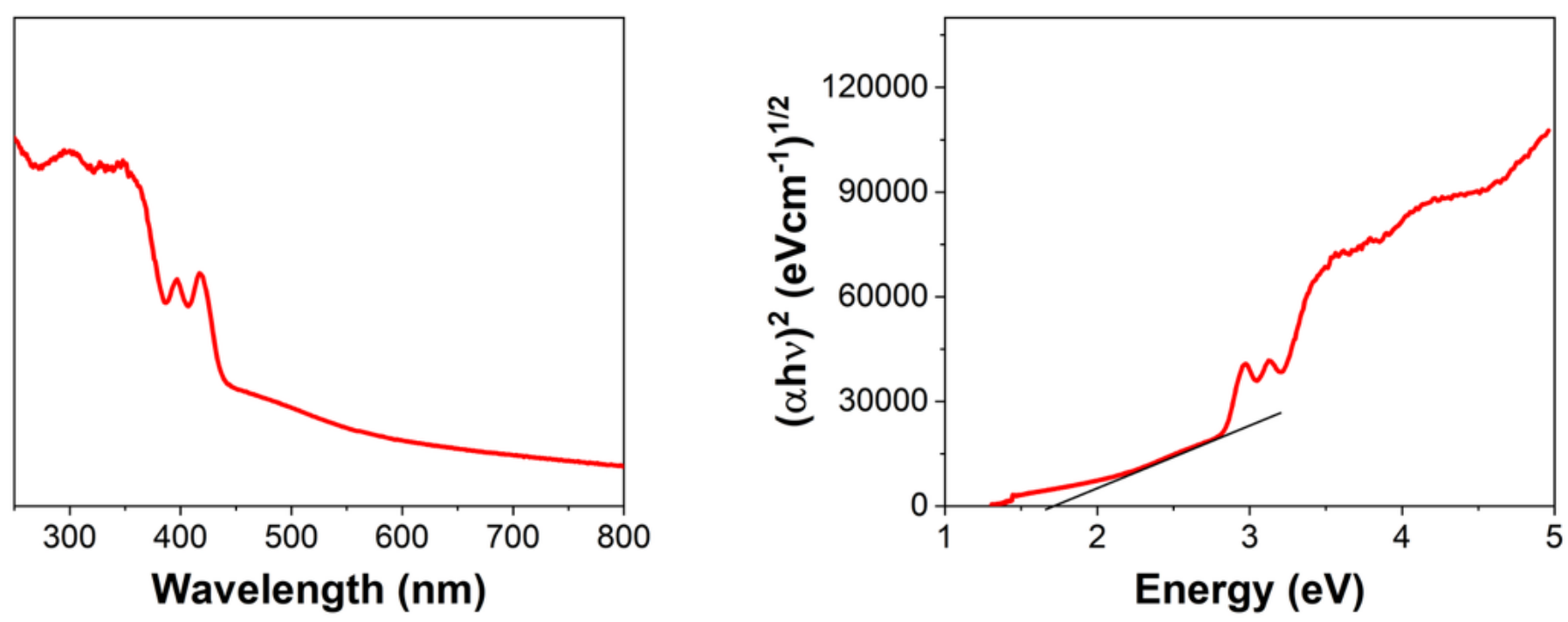

Figure S6. Solid-state UV−vis-NIR absorption spectra of *dia-c11* **Joa-COF-2**. (b) Tauc's plot (optical band gap = 1.73 eV).

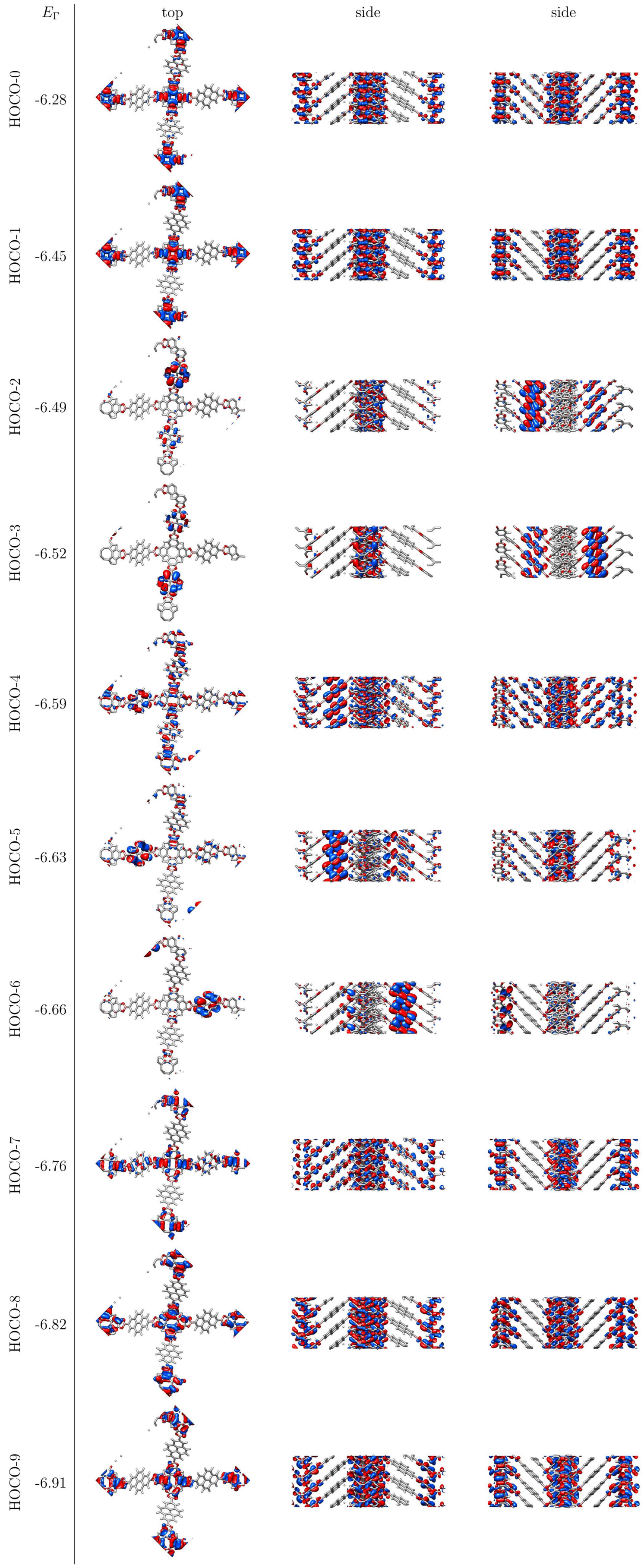

Figure S7. HOCO orbitals of *dia-c11* **Joa-COF-2** at the PBE level in real space.

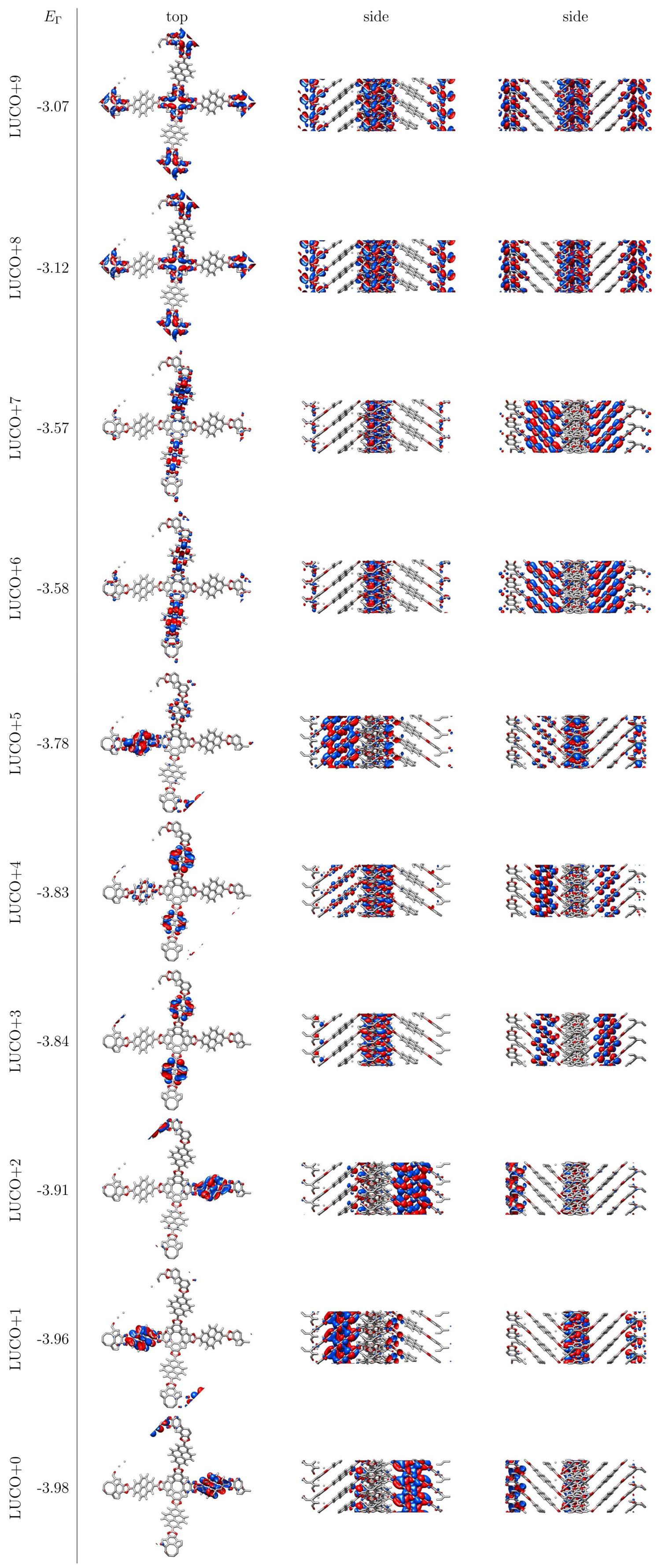

Figure S8. LUCO orbitals of *dia-c11* **Joa-COF-2** at the PBE level in real space.

Table S1. Pawley Fitting.

Parameters	Refined Values
a [Å]	24.47
b [Å]	27.26
c [Å]	4.69
α (°)	85.8
β (°)	85.2
γ (°)	85.2
Zero Point	0.018
Space Group	P1
R_{wp}	15.7
GOF	4.8
χ^2	23.4

Table S2. Structural parameters and computed porosities of *dia-c11* **Joa-COF-2**.

	a	b	c	alpha	beta	gamma	Pore limiting diameter	Maximal pore diameter	Surface Area	Pore access. Volume	Density
	Å	Å	Å	°	°	°	nm	nm	m²/g	cm³/g	g/cm³
poreblazer	23.44	26.81	4.69	85.9	85.35	85.17	0.8	0.9	1027	0.39	0.99
ZEO++							0.8	0.9	1277	0.41	0.99

Table S3. Key electronic properties computed with the PBE and B3LYP functionals, light basis set on a 2x2x7 k-point grid.

Hamiltonian	Gap	E_F
	eV	eV
PBE-MBD	2.140	-5.054
B3LYP	3.343	-5.113

General Procedures

Reagents for synthesis were, if not otherwise specified, purchased from Aldrich, TCI or Fluorochem. Column chromatography was carried out using Silica gel 60 (40-60 μm) from Scharlab. Analytical thin layer chromatography (TLC) was done using aluminum sheets (20x20 cm) pre-coated with silica gel RP-18W 60 F254 from Merck. UV-active compounds were detected with a UV-lamp from CAMAG at wavelength λ = 254 or 366 nm. Pyrene-2,7-diboronic acid[1] and 2,3,6,7,10,11,14,15-octamethoxytetraphenylene[2-3] were synthesized according to reported methods.

NMR spectra in solution were recorded on Bruker Avance 400 spectrometer at room temperature K using partially deuterated solvents as internal standards. Coupling constants (J) are denoted in Hz and chemical shifts (δ) in ppm. Multiplicities are denoted as follows: s = singlet, d = doublet, t = triplet, m = multiplet, br = broad.

Solid-State ^{13}C, ^{11}B and CP/MAS NMR spectra were recorded on a Bruker Avance III 400 MHz NMR spectrometer at a MAS rate of 12 kHz and a CP contact time of 2 ms.

Matrix Assisted Laser Desorption Ionization (coupled to a Time-Of-Flight analyzer) experiments (MALDI-TOF) were recorded on Bruker REFLEX spectrometer in POLYMAT.

ATR-FTIR spectra were recorded on a Bruker ALPHA ATR-IR spectrometer.

The powder X-ray diffraction (PXRD) patterns were collected by using a PHILIPS X'PERT PRO automatic diffractometer operating at 40 kV and 40 mA, in theta-theta configuration, secondary monochromator with Cu-Kα radiation (λ = 1.5418 Å) and a PIXcel solid state detector (active length in 2θ 3.347º). Data were collected from 1 to 50° 2θ (step size = 0.026 and time per step = 300 s, total time 40 min) at room temperature. A variable divergence slit, giving a constant 4.0 mm area of sample illumination, was used.

Nitrogen adsorption isotherms were measured at 77 K in a Micromeritics 3Flex. The samples were degassed in an Autosorb station at and 10^{-6} Torr at 100 °C prior to analysis. Specific surface area (SA) was calculated by multi-point Brunauer-Emmett-Teller (BET) method. Pore

size distributions were analysed by using the density functional theory (DFT) N2@77-Carbon slit pore model for the adsorption branch.

Electron Microscopy. TEM imaging was performed on a JEOL 2100F FEG-TEM microscope operated at 200 kV equipped with a Gatan K3-IS camera. EDS and EELS investigations were performed on a JEOL 2100+ TEM operated at 80 kV equipped with an Oxford Instruments X-MaxN 80 TLE EDS detector and Gatan Enfinium EELS detector.

Computer Models. Computer models were performed at the Density Functional Theory (DFT) level (PBE-MBD/light) with DFTB+[4-5] with the Fritz Haber Institute ab initio molecular simulations (FHI-aims) package.[6-8] For this, "light" or "light_194" numeric atomic orbitals, which approximately correspond to TZVP level of calculations were used, along with Many Body Dispersion (MBD-NL)[9-10] correction to account for van der Waals interactions. Band structure and band gap calculations were performed on at least 3x3x4 k-point grid. Effective masses were calculated with QuantumATK software suite[11] using default LCAO settings with a 2x2x2 k-point grid. Tight Binding methods were used to thoroughly explore the Potential Energy Surfaces of different **Joa-COF-2** model geometries, these calculations were performed with the DFTB+ program and Atomic Simulation Environment (ASE) suite.

Optical-pump THz-probe (OPTP) spectroscopy. The powder sample was compressed between two fused silica substrates to form a powder-based film. Driven by a commercial Ti:sapphire laser amplifier system, 800 nm laser pulses with a duration of ~50 fs and a repetition rate of 1 kHz were generated. The 400 nm pulsed laser used for photoexcitation was generated by frequency doubling the 800 nm pulsed laser through a BiB_3O_6 crystal. THz generation and detection were achieved by optical rectification and free-space electro-optic sampling, respectively. The measurements were performed in a dry N_2 environment at room temperature.

Synthetic Procedures

2,3,6,7,10,11,14,15-octahydroxytetraphenylene (1): *The synthesis was performed following a modified procedure described elsewhere, the spectroscopic data is consistent with that reported.*[12] A solution of 2,3,6,7,10,11,14,15-octamethoxytetraphenylene (50 mg, 0.92 mmol) in dry dichloromethane (2 mL) was cooled to –78 ºC and BBr_3 (0.2 mL) was added dropwise. The solution was stirred for 1 hour and it was warmed up to r.t. for 3 hours. The solvents were removed with a nitrogen flow; dry MeOH was added and removed again with a nitrogen flow. The resulting solid was filtered, washed with dichloromethane, and dried under vacuum to afford a white solid (Quantitative). ^{1}H NMR (400 MHz, DMSO-d_6) 6.34 (s, 8H). ^{13}C NMR (101 MHz, methanol-d_4) δ 144.65, 135.34, 117.10. EM (MALDI-TOF) (m/z): calculated for $C_{24}H_{16}O_8$: 432.08; found: 432.08 $[M]^+$.

Joa-COF-2: Tetraphenylene **1** (16 mg, 0.037 mmol) and pyrene-2,7-diboronic acid (21.6 mg, 0.074 mmol) were sonicated in 1.8 mL of a mixture of degassed *N*,*N*-dimethylacetamide/mesitylene (1:5) in a pre-scored 5 mL ampoule under nitrogen. The suspension was degassed by using three freeze-pump-thaw cycles. The ampoule was sealed off using flame and heated at 125 ºC for 3 days. The white precipitate was collected by filtration and washed five times with anhydrous tetrahydrofurane, acetone and hexane. The powder was dried at 30 ºC under vacuum for 24 hours. 23 mg (85 %) of **Joa-COF-2** were obtained.

References

[1] A. G. Crawford, Z. Liu, I. A. I. Mkhalid, M.-H. Thibault, N. Schwarz, G. Alcaraz, A. Steffen, J. C. Collings, A. S. Batsanov, J. A. K. Howard, T. B. Marder, *Chem. Eur. J.* **2012**, *18*, 5022-5035.

[2] S. N. Talapaneni, J. Kim, S. H. Je, O. Buyukcakir, J. Oh, A. Coskun, *J. Mater. Chem. A* **2017**, *5*, 12080-12085.

[3] X. Li, J. W. Han, H. N. C. Wong, *Asian J. Org. Chem.* **2016**, *5*, 74-81.

[4] B. Hourahine, B. Aradi, V. Blum, F. Bonafé, A. Buccheri, C. Camacho, C. Cevallos, M. Y. Deshaye, T. Dumitrică, A. Dominguez, S. Ehlert, M. Elstner, T. v. d. Heide, J. Hermann, S. Irle, J. J. Kranz, C. Köhler, T. Kowalczyk, T. Kubař, I. S. Lee, V. Lutsker, R. J. Maurer, S. K. Min, I. Mitchell, C. Negre, T. A. Niehaus, A. M. N. Niklasson, A. J. Page, A. Pecchia, G. Penazzi, M. P. Persson, J. Řezáč, C. G. Sánchez, M. Sternberg, M. Stöhr, F. Stuckenberg, A. Tkatchenko, V. W.-z. Yu, T. Frauenheim, *J. Chem. Phys.* **2020**, *152*, 124101.

[5] B. Aradi, B. Hourahine, T. Frauenheim, *J. Phys. Chem. A* **2007**, *111*, 5678-5684.

[6] V. Blum, R. Gehrke, F. Hanke, P. Havu, V. Havu, X. Ren, K. Reuter, M. Scheffler, *Comput. Phys. Commun.* **2009**, *180*, 2175-2196.

[7] A. Marek, V. Blum, R. Johanni, V. Havu, B. Lang, T. Auckenthaler, A. Heinecke, H. J. Bungartz, H. Lederer, *J. Phys. Condens. Matter.* **2014**, *26*, 213201.

[8] V. W.-z. Yu, F. Corsetti, A. García, W. P. Huhn, M. Jacquelin, W. Jia, B. Lange, L. Lin, J. Lu, W. Mi, A. Seifitokaldani, Á. Vázquez-Mayagoitia, C. Yang, H. Yang, V. Blum, *Comput. Phys. Commun.* **2018**, *222*, 267-285.

[9] A. Ambrosetti, A. M. Reilly, R. A. DiStasio, Jr., A. Tkatchenko, *J. Chem. Phys.* **2014**, *140*, 18A508.

[10] J. Hermann, A. Tkatchenko, *Phys. Rev. Lett.* **2020**, *124*, 146401.

[11] S. Smidstrup, T. Markussen, P. Vancraeyveld, J. Wellendorff, J. Schneider, T. Gunst, B. Verstichel, D. Stradi, P. A. Khomyakov, U. G. Vej-Hansen, M.-E. Lee, S. T. Chill, F. Rasmussen, G. Penazzi, F. Corsetti, A. Ojanperä, K. Jensen, M. L. N. Palsgaard, U. Martinez, A. Blom, M. Brandbyge, K. Stokbro, *J. Phys. Condens. Matter.* **2020**, *32*, 015901.

[12] T. Ashirov, K. S. Song, A. Coskun, *ACS Appl. Nano Mater.* **2022**, *5*, 13711-13719.
